# Cellular heterogeneity in pancreatic cancer: the different faces of gremlin action

**DOI:** 10.1038/s41392-022-01203-8

**Published:** 2022-10-12

**Authors:** Aristidis Moustakas, J. Matthias Löhr, Rainer L. Heuchel

**Affiliations:** 1 Department of Medical Biochemistry and Microbiology, Uppsala University, Uppsala, Sweden; 2 Department of Clinical Intervention and Technology (CLINTEC), Karolinska Institutet, Stockholm, Sweden

**Keywords:** Gastrointestinal cancer, Tumour heterogeneity

A recent publication in Nature by Lan et al., presented GREM1 (gremlin 1) as an important regulatory node of cellular plasticity in pancreatic ductal adenocarcinoma (PDAC).^1^ Knockout experiments in mice point to an important role of *Grem1* in retaining an epithelial, differentiated phenotype of pancreatic cancer cells presumably by inhibiting BMP signalling.

The starting idea had been to comparatively analyse cells from *Kras*^*LSL-G12D/+*^;*Trp53*^*fl/fl*^;*Pdx1-cre;Rosa26*^*LSL-YFP*^ (KPCY) mouse PDAC tumours after cell sorting into YFP^+^;Epcam^high^ epithelial and YFP^+^;Epcam^low^ cells, followed by mRNA-expression analysis of epithelial and mesenchymal marker genes. Epcam^high^ cells predominantly expressed epithelial markers like cytokeratin19 (CK19), as expected, while Epcam^low^ cells showed highly enriched expression of hallmark genes for epithelial-to-mesenchymal transition (EMT). Among them was *Grem1*, which was expressed 30-times higher in Epcam^low^ compared to Epcam^high^ cells. Grem1 is a secreted glycoprotein that acts as an antagonist to bone morphogenetic protein (BMP) family ligands. Grem1 has also been identified to act as a stemness factor in diverse epithelial and brain tumours, a function that may or may not be directly linked to regulation of BMP signalling.^2^

Interestingly, gene set enrichment analysis of Grem1^high^ and Grem1^low^ PDACs identified in The Cancer Genome Atlas showed enriched expression of EMT hallmark genes especially in Grem1^high^ PDACs. To elucidate the role of *Grem1* in affecting the cellular plasticity between epithelial and mesenchymal PDAC phenotypes, *Grem1* knockout was induced in the background of an established pancreatic cancer dual recombinase mouse model driven by *Kras*^*G12D*^;*Trp53*^*knockout*^. This resulted in poorly differentiated, more mesenchymal tumours containing predominantly vimentin^+^ (Vim^+^) mesenchymal-like PDAC cells, less epithelial-like CK19^+^ glandular PDAC cells and a significantly higher number of CK19^+^Vim^+^ hybrid EMT cancer cells. In support of these observations, the authors analysed subcutaneous tumours from injected fluorescently labelled cells derived from *Kras*^*G12D*^;*Trp53*^*knockout*^ organoids at several time points following a five day induction of the conditional *Grem1*^*knockout*^ allele. This kinetic experiment demonstrated that the ratio of epithelial-like versus mesenchymal-like cells clearly shifted in favour of a more mesenchymal cell phenotype of PDAC over time. The authors interpret this as a phenotypic shift of CK19^+^Vim^+^ cells to CK19^−^Vim^+^ cells via EMT. The criteria for PDAC grading in the WHO Classification of Tumours of the Digestive System, take among others into account the grade of de-differentiation and number of mitoses in the higher-grade areas of the tumour. Since at the timepoint of *Grem1*^*knockout*^ induction, CK19^-^Vim^+^ and Vim^+^ PDAC cells already existed, although in lower numbers, it cannot be excluded that the higher proliferation rate of these cells also contributed to the more mesenchymal appearance of the *Grem1*^*knockout*^ tumours. Using another mouse model, based on pancreas-specific *Kras*^*G12D*^ oncogenic mutation but heterozygosity of the *Trp53* null allele (*Trp53*^*het*^), the loss of *Grem1* in already established PDAC tumours resulted in significantly higher numbers of liver and lung metastases compared to *Grem1*^*wt*^ control mice, supporting an important role of *Grem1* in suppression of metastasis formation via EMT.

Among the genes with enriched expression in Epcam^low^ PDAC cells the authors found, besides *Grem1* and *BMP2* other SMAD-binding/BMP target genes like *ID3/4*. To elucidate the relationship between *Grem1* and BMP signalling, cell culture experiments were performed on organoids derived from their PDAC mouse models. In these experiments, genetic ablation of *Grem1* as well as chemical stimulation/inhibition with BMP2/LDN19318 (inhibitor of BMP type I receptor kinases) showed increased Smad1/5/9 phosphorylation. The EMT transcription factors Snail and Slug were identified as target genes of BMP signalling with functional importance for the mesenchymal PDAC phenotype. Mechanistically, BMP Smad signalling could induce transcription of Snail, which can then induce Slug transcription, as previously delineated in non-pancreatic tumour cells.

**Fig. 1:**
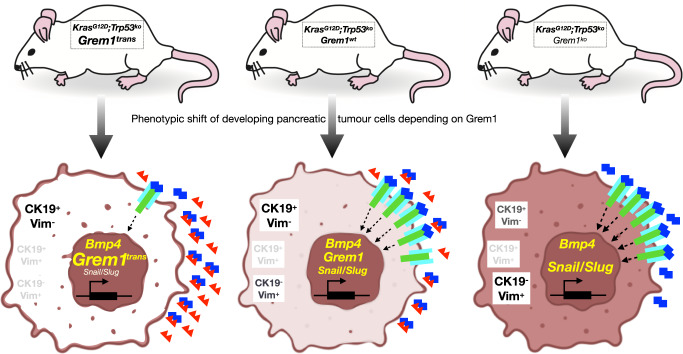
Change in cellular heterogeneity according to phenotypic shift of developing pancreatic tumours (*Kras*^*G12D*^;*Trp53*^*knockout*^) depending on the presence of Grem1. The schematic cartoon highlights the shift from a predominantly epithelial, glandular tumour type containing predominantly CK19^+^Vim^−^ PDAC cells (left) to a predominantly mesenchymal, invasive pancreatic tumour type containing predominantly CK19^−^Vim^+^ PDAC cells (right) depending on the expression of Grem1 (red triangles). Bmp2 (blue squares) activating the respective Bmp receptors (light blue/green), found highly expressed in more mesenchymal (Vim^+^) PDAC cells, up-regulates Grem1 expression which in turn can bind to and partially inhibit Bmp signalling via negative feedback (middle part). The phenotypic shift is indicated by gradual darkening of the schematic tumour cells (lower part). The amount of the different cellular phenotypes found in the respective tumours are indicated by letter size and -grey scale. The level of *Grem1*, *Snail* and *Slug* expression are likewise indicated by size and colour code. *Kras*^*G12D*^, oncogenic allele of *Kras* with pancreas-specific expression; *Grem1*^*trans*^, transgenic over-expression of *Grem1*; *Grem1*^*wt*^, wildtype allele of *Grem1*; *Grem1*^*ko*^, pancreas-specific knockout of *Grem1*

Cancers, and especially PDAC are very heterogeneous, i.e., they develop multiple clones within the primary tumour over time, whose mutational gene signatures are also reflected in the metastases formed thereof. Furthermore, PDAC is an exceptionally desmoplastic/fibrotic, stroma-rich cancer type, that has been roughly stratified into two molecular subtypes, classical-pancreatic/progenitor and squamous/basal-like/quasi-mesenchymal based on transcriptomic profiling.^3^ But not only the PDAC cells themselves display strong heterogeneity, but also the surrounding tumour stroma, containing tumour suppressive myofibroblastic cancer-associated fibroblasts (myCAFs) in the direct vicinity of more glandular PDAC cells and tumour-supportive inflammatory iCAFs relatively more distant from the PDAC cells.^4^ Interestingly, the authors not only find α-smooth muscle actin/Acta2-positive myCAFs expressing significantly higher levels of *Grem1*-mRNA than iCAFs, but they also observed a considerably higher number of *Acta2*^+^-myCAFs in *KPFC/Grem1*^*wt*^ compared to *KPFC/Grem1*^*knockout*^-tumours. Therefore, it might also be possible that *Grem1*-expressing myCAFs are contributing to maintain the neighbouring glandular PDAC cells in their epithelial, differentiated state (Fig. 1). This is reminiscent of studies on intestinal regeneration, where GREM1-secreting cells were mapped as being mesenchymal cells, layered at the bottom of the intestinal crypts.^5^

So, how can *Grem1* act as a gatekeeper of the epithelial state? The experimental results tell that knockout of *Grem1* in PDAC cells leads to more de-differentiated tumours of mesenchymal-type, whereas over-expression of *Grem1* leads to more differentiated tumours. Mesenchymal-type PDAC cells express high levels of *Grem1* and *BMP4* mRNA. The actual expression of the respective proteins was not analysed, which would be of interest to measure or estimate the stoichiometry and with that the effectiveness of the net signalling outcome generated by the BMP-Grem1 equilibrium. Furthermore, analysis of the mechanism of GREMLIN protein transport from mesenchymal to epithelial cell types within the tumour remains to be explained (Fig. 1). The provided experimental data also do not prove that the BMP-Grem1 axis is a direct driver of EMT/MET plasticity. Grem1 might act by antagonising alternative ligands of the BMP/transforming growth factor β (TGFβ) families or even via more direct signalling routes. The lack of *Grem1* in mesenchymal-type PDAC cells could result in a higher proliferation rate of these cells without affecting plasticity. Higher proliferation rate is one characteristic of high grade PDAC cells. Finally, it needs to be mentioned that TGFβ plays a major role also with regard to cellular plasticity for both, the pancreatic cancer cells and the surrounding microenvironmental cells, which has not been touched upon by the current study.^6^ For example, TGFβ not only drives EMT of PDAC cells in collaboration with Kras, but is also a major profibrogenic driver of the characteristic desmoplasia as well as a major immunosuppressive factor.

The Lan et al. paper emphasises the established role of GREMLIN as a key determinant of epithelial and stem-like phenotypes in various human tumours and suggests the usefulness of agonistic GREMLIN-like biologicals that could possibly mediate differentiation therapy in human tumours, including PDAC. If such biologicals develop, the impact of the current study on the future of novel treatments of pancreatic and other cancer types, will prove to be high. Until then, however, further experiments are required regarding the exact identification of the source and target cells of Grem1 protein. Another important question is about the consequence(s) of Grem1 protein binding to these target cells, such as block or reversion of EMT, or preferential proliferation of more epithelial-like cancer cells.
